# miR-210 and miR-152 as Biomarkers by Liquid Biopsy in Invasive Ductal Carcinoma

**DOI:** 10.3390/jpm11010031

**Published:** 2021-01-06

**Authors:** Beatriz C. Lopes, Cristine Z. Braga, Fabrício V. Ventura, Jéssica G. de Oliveira, Edson M. Kato-Junior, Newton A. Bordin-Junior, Debora A. P. C. Zuccari

**Affiliations:** 1 Institute of Biosciences, Letters and Exact Sciences (IBILCE), Sao Jose do Rio Preto, Sao Paulo 15054-000, Brazil; beatrizclbio@gmail.com; 2 Faculdade de Medicina de Sao Jose do Rio Preto (FAMERP), Sao Jose do Rio Preto, Sao Paulo 15090-000, Brazil; cris_zamp@hotmail.com (C.Z.B.); ventura.bio.med@gmail.com (F.V.V.); jessicag.oliveira21@outlook.com (J.G.d.O.); edsonkato_jr@hotmail.com (E.M.K.-J.); 3 Hospital de Base de Sao Jose do Rio Preto, Sao Jose do Rio Preto, Sao Paulo 15090-000, Brazil; bordinjunior@gmail.com; 4 Laboratory of Molecular Investigation of Cancer (LIMC), Avenue Brigadeiro Faria Lima, 5416, Vila Sao Pedro, Sao Jose do Rio Preto, Sao Paulo 15090-000, Brazil

**Keywords:** breast cancer, miRNAs, liquid biopsy, angiogenesis, biomarkers, early diagnosis

## Abstract

Detecting circulating microRNAs (miRNAs; miRs) by means of liquid biopsy is an important tool for the early diagnosis and prognosis of breast cancer (BC). We aimed to identify and validate miR-210 and miR-152 as non-invasive circulating biomarkers, for the diagnosis and staging of BC patients, confirming their involvement in tumor angiogenesis. Methods: RT-qPCR was performed and MiRNA expression analysis was obtained from plasma and fragments of BC and benign breast condition (BBC) women patients, plus healthy subjects. Additionally, the immunohistochemistry technique was carried out to analyze the expression of target proteins. Results: Tumor fragments showed increased expression of oncomiR-210 and decreased expression of miR-152 tumoral suppressor. Both miRNAs were increased in plasma samples from BC patients. The receiver operating characteristic (ROC) curve analysis revealed that only the expression of oncomiR-210 in tissue samples and only the expression of the miR-152 suppressor in plasma have the appropriate sensitivity and specificity for use as differential biomarkers between early/intermediate and advanced stages of BC patients. In addition, there was an increase in the expression of hypoxia-inducible factor 1-alpha (HIF-1α), insulin-like growth factor 1 receptor (IGF-1R), and vascular endothelial growth factor (VEGF) in BC patients. On the contrary, a decrease in Von Hippel–Lindau (VHL) protein expression was observed. Conclusions: This study showed that increased levels of miR-210 and decreased levels of miR152, in addition to the expressions of their target proteins, could indicate, respectively, the oncogenic and tumor suppressive role of these miRNAs in fragments. Both miRNAs are potential diagnostic biomarkers for BC by liquid biopsy. In addition, miR-152 proved to be a promising biomarker for disease staging.

## 1. Introduction

The high mortality rate for breast cancer (BC) is mainly related to the development of metastasis, a process that depends on angiogenesis [1]. Data show that this malignant evolution is aggravated, in most cases, by the late diagnosis of the disease [2]. Therefore, the identification of biomarkers that can diagnose cancer in the early stage and predict the behavior of the tumor is of special interest to the patients.

As the tumor grows, there is a reduction in oxygen at the center of the tumor, creating adverse conditions of hypoxia, which induces increased expression of numerous pro-angiogenic factors, such as hypoxia-inducible factor 1-alpha (HIF-1α) and insulin-like growth factor type 1 receptor (IGF-1R) [3]. Increased levels of HIF-1α and IGF-1R stimulate vascular endothelial growth factor (VEGF) expression [4]. On the contrary, anti-angiogenic factors, such as Von Hippel–Lindau (VHL) protein act in the degradation of HIF-1α and, consequently, decrease the expression of VEGF [5].

Currently, liquid biopsy has been gaining ground as a promising tool for the detection of neoplasms, having the benefit of being less invasive and causing minimal discomfort and risk to patients. This procedure consists of the collection of any bodily fluid for the purpose of analyzing information from the tumor, including circulating nucleic acids—circulating tumor DNA (ctDNA) and circulating tumor RNA (ctRNA) [6].

These circulating biomarkers have advantages over tissue biomarkers as they are easily obtained and can also be monitored routinely, resulting in real-time detection, and consequently effective diagnosis and prognosis. With the advancement of this technique, a growing series of research studies have been developed for the purpose of discovering new circulating biomarkers that are more sensitive, characterizing specific subtypes of each tumor type [7].

Additionally, a class of small non-coding RNAs, called microRNAs (miRNAs; miRs), has been studied as potential biomarkers in cancer. MiRNAs are small, non-coding endogenous RNAs with approximately 22 nucleotides that control numerous cell pathways through the regulation of gene expression in the post-transcriptional phase [8,9].

A broad review showed the most significant miRNAs for BC and which regulators (hallmarks) they involved [10]. Among them, miR-210 is considered an oncogenic miRNA which exhibits regulation mediated by HIF-1α and VHL. HIF-1α promotes increased expression of miR-210 and this leads to stabilization of HIF-1α, suggesting that both miRNA and its target gene are involved in a positive feedback loop. HIF-1α, in turn, will stimulate the expression of genes that promote angiogenesis, such as VEGF [11,12,13]. In addition, studies have shown that miR-210 expression was significantly higher in patients with BC in the preoperative phase compared to women who underwent tumor removal surgery [14]. Moreover, a study showed that elevated levels of miR-210 lead to increased tumor progression and migration and reduced cell cycle arrest in MCF-7 breast tumor cells [15].

Another miRNA present in the review by Bertoli, Cava, and Castiglioni is miR-152, which belongs to the miR-148/152 family. This miRNA is involved in the regulation of angiogenesis, being considered a tumor suppressor in BC [10]. Studies investigated the regulation of the IGF-1R receptor by miR-152. The high expression of this miR inhibits the expression of IGF-1R through its binding to the 3′-UTR region, leading to the blocking of the expression of HIF-1α and VEGF [16,17]. In BC, miR-152 showed low levels of expression in tumor tissue compared to adjacent non-tumor tissue, as well as in a series of BC cell lines [18,19].

**Figure 1:**
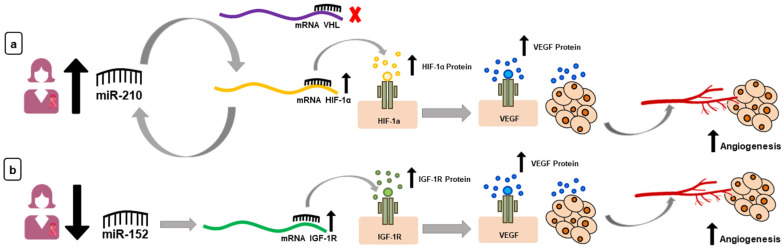
Candidate microRNAs (miRNAs) and their target proteins. (**a**) High levels of miR-210 in women with breast cancer (BC) leads to increased levels of hypoxia-inducible factor 1-alpha (HIF-1α) (activates a looping cascade) and, in addition, there is no blocking of HIF-1α by Von Hippel–Lindau (VHL) protein. Elevated levels of HIF-1α lead to an increase in vascular endothelial growth factor (VEGF). (**b**) With regard to miR-152, there is a low expression of this miRNA in women with BC, which leads to the increase of the IGF-1R protein and, later, of the VEGF protein.

In summary, on tumor progression, the high expression of oncomiR-210 and the low expression of the miR-152 suppressor trigger the increase in VEGF. The mechanisms of action of both miRNAs in the angiogenic pathway are summarized in Figure 1.

In recent years, studies have shown that miRNAs are not only detected in tissues, but also in fluids [9]. The circulating miRNAs, that represent the miRNA population in the free portion of body fluids, have attracted interest in the field of biomarker discovery because they are involved in several gene regulation processes [20]. Therefore, it is of great importance for patients to validate tumor biomarkers that are capable of diagnosing and predicting the staging for BC and can still be detected by a non-invasive method such as liquid biopsy. The aim of this study was to validate two miRNAs involved in the angiogenic process, and to evaluate the potential of these as biomarkers of diagnosis and staging in BC.

## 2. Materials and Methods

This study was approved by the Research Ethics Committee (CEP) of the Faculty of Medicine of Sao Jose do Rio Preto (FAMERP), Sao Jose do Rio Preto, Brazil, #2.118.866/2017, and it was elaborated following the national and international rules of ethics in experiments with human samples.

### 2.1. Experimental Groups

The samples were collected from patients at their first medical appointment and selected by BI-RADS™ that indicated a higher probability of BC (4C and 5). Of the total samples, 30 women from the BC group and 5 from the benign breast condition (BBC) group were selected, based on the inclusion criteria pre-established in this study. The experimental groups of this study were as follows:

- Group Breast Cancer (BC): women with breast cancer (plasma/fragment: *n* = 30);
- Group Benign Breast Conditions (BBC): women with benign breast conditions (plasma/fragment: *n* = 5);
- Control: women with no atypical breast, and no history of BC in the family (plasma: *n* = 15; fragment: *n* = 5).

Inclusion criteria for BC: patients pathologically diagnosed with BC who had undergone surgical treatment; exclusion criteria for BC: patients who had received radiotherapy and chemotherapy before specimens were taken; exclusion criteria for all groups: women with other severe organ diseases; patients with any inflammatory process at the time of collection. All collections were performed at the Base Hospital, Sao Jose do Rio Preto, Brazil.

### 2.2. Collections

With respect to groups BC and BBC, the fragments were obtained by core biopsy performed. For the control group, the mammary fragments were obtained in mammoplasty reduction. Blood samples were collected by venipuncture (up to 5 mL per patient) and processed within one hour of collection.

### 2.3. Determination of Diagnosis and Pathological Prognosis Staging

All BC and BBC women included in this study had their diagnoses confirmed by the pathology team at the Base Hospital—Sao Jose do Rio Preto, SP/Brazil. To determine the staging of each patient, the most recent version (8th edition/2018) published by the American Joint Committee on Cancer (AJCC) was used.

The present study divided the women with staging Ia, Ib, IIa, and IIb and considered the respective patients to have an early/intermediatestage. Women with staging IIIa, IIIb, IIIc, and IV were considered to have an advanced stage.

### 2.4. Analysis of miRNA Expression by RT-qPCR

Plasma RNA extraction was performed with the miRNeasy Serum/Plasma Advanced kit (Qiagen, CA, USA) (200 μL plasma), and the Trizol™ reagent (Life Technologies, Carlsbad, CA, USA) was used for the fragments. The RNA concentration and purity was assessed using a spectrophotometer (NanoDrop™ 2000/2000c, Thermo Fisher Scientific, Waltham, MA, USA). The status purity was verified using A260/A280 ratios (range: 1.8–2.0). The complementary DNA (cDNA) was obtained by reverse transcriptase technique from the RNA extracted from the samples (50 ng). cDNA was synthesized from 50 ng of total RNA with the TaqMan™ MicroRNA Reverse Transcription kit (Applied Biosystems, Foster City, CA, USA).

The quantitative real-time polymerase chain reaction (qPCR) was performed using the StepOnePlus™ Real-Time PCR System (Applied Biosystems). Each sample were normalized against U6 levels. Reactions for miRNA expression analysis (and the reference gene, U6) were performed in triplicate (plasma) and duplicate (fragment) using 10 μL of 2 × TaqMan^®^ Gene Expression Master Mix (Applied Biosystems), 1 μL of 20 × TaqMan Gene Expression Assay (Applied Biosystems), 3 μL cDNA by volume, and 6 μL of H2O DEPC, at a final volume of 20 μL.

The expression values of the miRNAs of interest were determined by the relative quantification (RQ) method in relation to normalizing of the gene used as reference (2−ΔΔCq). Details of the sequences of miRNAs (miR-210 and miR-152) are available in Table S1, Supplementary Materials.

### 2.5. Analysis of Target Protein Expression by Immunohistochemistry

The protein expression of the target genes HIF-1α, IGF-IR, VHL, and VEGF was performed by immunohistochemistry assay, and their standardization followed the instructions provided by the manufacturer. Histological sections of 4 μm were obtained from paraffin-embedded material and adhered to silanized slides. The deparaffinization was performed on xylol followed by hydration, and subsequently incubated with hydrogen peroxide with the blocking of proteins (blocking non-specific reactions). The material was incubated with the primary antibody of each specific protein for 18 h at 4 °C. The Complement and HRP Conjugate (REVEAL™ Biotin-Free Polyvalent DAB - Spring Bioscience, Pleasanton, CA, USA) were applied, followed by the chromogenic substrate (DAB) and Harris hematoxylin.

All immunoreactions were accompanied by a positive control and a negative control (without primary antibody). The slides were observed under a 40× objective of the Olympus BX60 light equipment (Olympus, Shinjuku, Tokyo, Japan) and analyzed by optical densitometry. For each sample, 3 different fields were photographed only in the immunoreactive areas, in which 20 points were quantified with ImageJ software, totaling 60 quantified points for each slide. The values were obtained in arbitrary units (a.u.) and demonstrated the average optical density (DOM) for each sample. The details of the antibodies and the photomicrographs of the positive and negative controls are presented in Table S2 and Figure S1, respectively, Supplementary Materials.

### 2.6. Statistical Analysis

The results were submitted to descriptive analysis to determine normality. For each group, the Gaussian distribution test or D’Agostino and Pearson omnibus normality test was applied. The comparison of two parameters was performed using the Mann–Whitney test, and for three or more parameters the Kruskal–Wallis test (posteriorly Dunn post test) was performed. Data were presented as mean ± standard error of the mean (SEM). Values of *p* < 0.05 were considered significant and all analyses were performed using GraphPad Prism 8 software (GraphPad Software, Inc., San Diego, CA, USA). In order to measure the diagnostic accuracy of each miRNA, the receiver operating characteristic (ROC) curve was used. In addition, the sensitivity and specificity of the optimum cutoff point were defined as those values that maximized the area under the curve (AUC).

## 3. Results

### 3.1. Characterization of Study Population

For the BC group, the demographic characteristics analyzed were age, distant metastasis, pathological prognosis staging, histological grade, and expression of hormonal receptors (ER, RP, HER2). Regarding age, among the 30 women included in this study, 25 (83.33%) had an age greater than or equal to 50 years, the mean age being 59 years (29–79 years). For the pathological prognosis staging, 14 samples (46.66%) were of the early/intermediate stages (Ia, Ib, IIa, and IIb), and 16 samples (53.34%) were of the advanced stage (IIIa, IIIb, IIIc, and IV). The histological grade of the tumor showed that 3 (10%), 20 (66.67%), and 7 (23.33%) samples were tumors that were low differentiated (Grade I), moderately differentiated (Grade II), and well differentiated (Grade III), respectively. Distant metastasis was found in 6 (20%) women with metastatic BC at the time of this study. For the expression of hormone receptors, the data show that 22 (73.33%), 19 (63.33%), and 14 (46.67%) samples were positive for ER, PR, and HER2, respectively. The mean age of the BBC group was 52 years (40–70 years), and the mean age of the control group was 49 years (23–70 years).

### 3.2. Expression Levels of miRNAs in Breast Cancer

**Figure 2:**
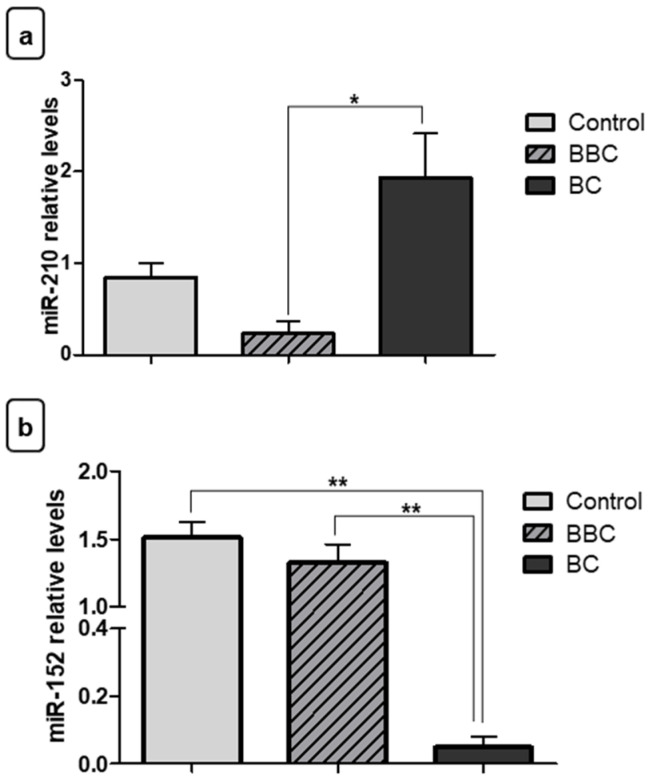
Evaluation of miRNA expression in fragments as diagnostic biomarkers. (**a**) Relative quantification showed that miR-210 exhibited increased expression in samples of tumor fragments from patients with breast cancer (BC) vs. those with benign breast conditions (BBC). (**b**) Contrastingly, miR-152 showed decreased expression in samples of BC fragments compared to the control group and to the BBC group. Significant value in Kruskal–Wallis and Dunn’s post test (±SEM - standard error of the mean; * *p* < 0.05; ** *p* < 0.01).

Initially, we verified the expression of miR-210 and miR-152 in tumor fragments (BC), benign (BBC), and control to test the different levels of expression of these miRNAs in BC. MiR-210 showed increased expression in tumor fragments of BC compared to the BBC (* *p* < 0.05) (Figure 2a). However, there was no significant difference between BC fragments compared to the control group and between the BBC group and the control group. For miR-152, relative quantification showed decreased expression in tumor fragments of BC when compared to BBC fragments (** *p* < 0.01) and normal fragments (** *p* < 0.01) (Figure 2b).

**Figure 3:**
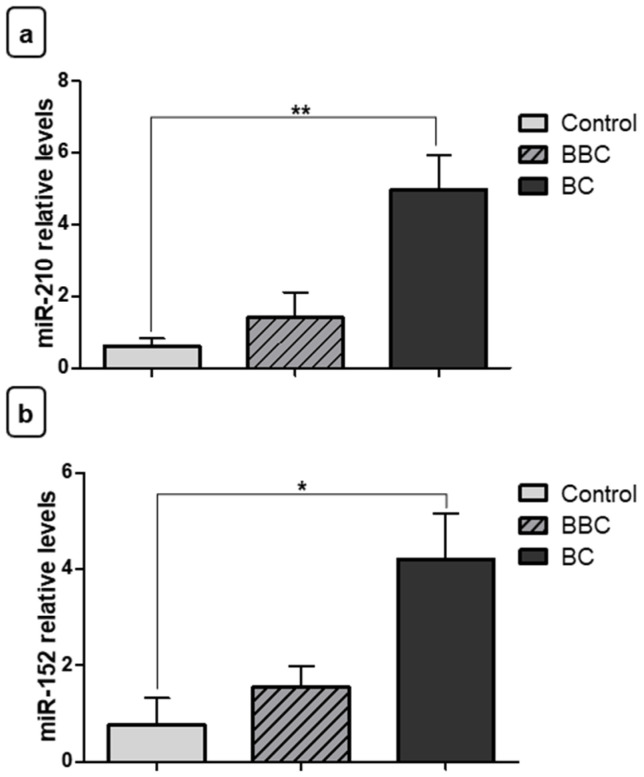
Evaluation of plasma miRNA expression as diagnostic biomarkers. (**a**) Relative quantification revealed that miR-210 showed increased expression in plasma samples from breast cancer (BC) patients vs. the control group. (**b**) The miR-152 also showed increased expression in plasma samples from women with BC vs. the control group. No significant difference was observed between the BC group and benign breast conditions (BBC) for any miRNA. Significant value in Kruskal–Wallis and Dunn’s post test (±SEM - standard error of the mean; * *p* < 0.05, ** *p* < 0.01).

Subsequently, we verified the expression levels of these miRNAs in plasma samples for the three different groups. With respect to miR-210, the relative quantification showed increased plasma expression for women with BC compared to the control group (** *p* < 0.01) (Figure 3a). However, there was no significant difference in plasma samples from the BC group compared to the BBC group or between the BBC group and the control group.

Surprisingly, we found that the tumor suppressor, miR-152, also had elevated levels in plasma samples from BC patients compared to the control group (* *p* < 0.05), different from the tumor fragments (Figure 3b). There was no significant difference in these miRs when compared to the BC group and women with BBC.

**Figure 4:**
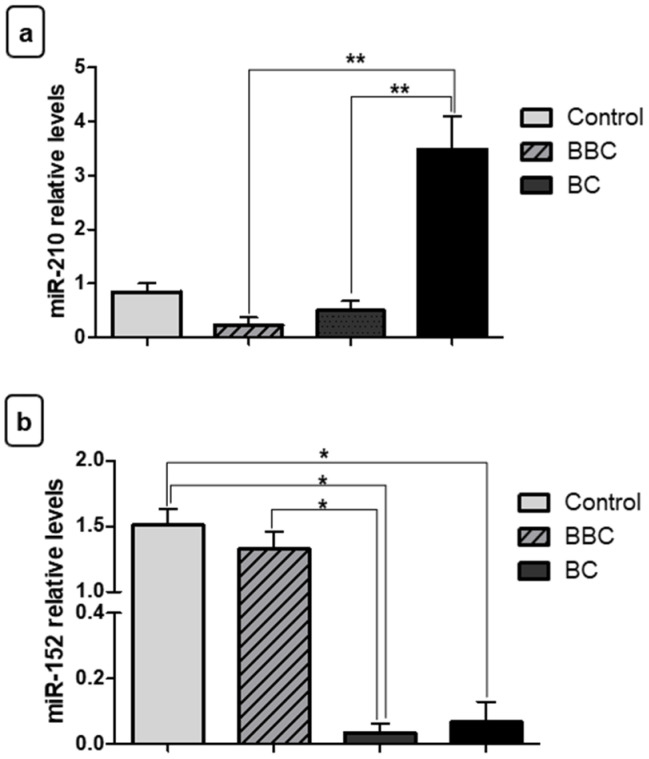
Evaluation of miRNA expression in fragments as staging biomarkers. (**a**) Relative quantification showed that there was increased expression of miR-210 in samples of breast cancer (BC) fragments with advanced stage when compared to women with early/intermediatestage of BC. Moreover, the increase was also significant with benign breast conditions (BBC). (**b**) miR-152 showed a significant decrease of expression in fragments from women with advanced stage of BC vs. the control group, and also decreased expression in early/intermediatestage malignant neoplasms when compared to women with BBC and the control group. There was no significant difference between the two stages’ groups. Significant value in Kruskal–Wallis and Dunn’s post test (±SEM - standard error of the mean; * *p* < 0.05; ** *p* < 0.01).

After finding the expression levels in the BC, BBC, and control groups, the miRNA expression levels were also analyzed in the staging groups. Our results show that miR-210 had increased expression in fragments of malignant neoplasms of advanced stage when compared to samples of early/intermediate stage BC fragments (** *p* < 0.01). In addition, there was increased expression in malignant neoplasms of advanced stage when compared to samples of BBC fragments (** *p* < 0.01) (Figure 4a). Regarding miR-152, relative quantification showed decreased expression in malignant neoplasm fragments of advanced stage in relation to the control group (* *p* < 0.05). The difference was also significant among women with early/intermediatestage malignant neoplasm vs. women with BBC (* *p* < 0.05) and control women (* *p* < 0.05) (Figure 4b). However, there was no difference between the different stages of BC for miR-152 in fragments.

**Figure 5:**
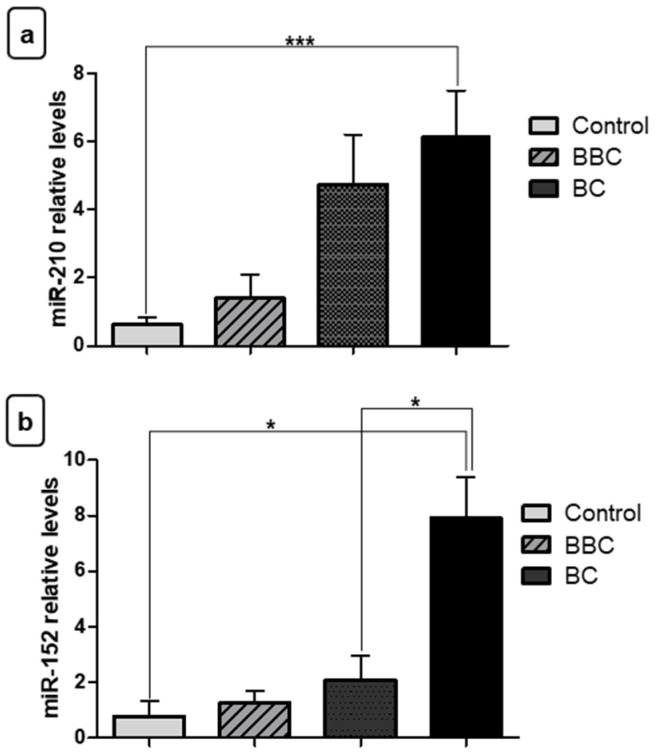
Evaluation of plasma miRNA expression as staging biomarkers. (**a**) Relative quantification showed that there was increased expression of miR-210 in plasma samples from women with breast cancer (BC) of advanced stage vs. controls. (**b**) With regard to miR-152, this showed increased expression in plasma samples from women with malignant neoplasms of advanced stage vs. women with early/intermediate stage and controls. Significant value in Kruskal–Wallis and Dunn’s post test (±SEM - standard error of the mean; * *p* < 0.05; *** *p* < 0.01). BBC: benign breast conditions.

For plasma samples, with respect to the miR-210, relative quantification showed increased expression in women with advanced stage of BC in relation to women controls (*** *p* < 0.001) (Figure 5a). However, there was no significant difference between the different stages of BC. Surprisingly, in addition to the increased expression level of the tumor suppressor, miR-152, in plasma of women with BC, this was also able to differentiate women with advanced stage from women with early/intermediate stage of BC and control women (* *p* < 0.05) (Figure 5b).

**Figure 6:**
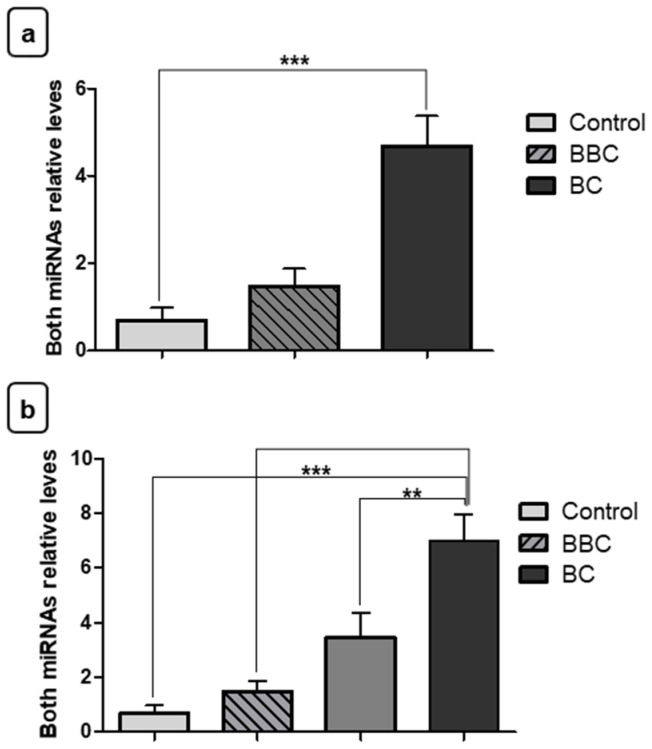
Evaluation of both miRNAs’ plasma expression as staging biomarkers. (**a**) Relative quantification showed that there was increased expression of both miRNAs in plasma samples from women with breast cancer (BC) vs. the control group. (**b**) Relative quantification showed that there was increased expression of both miRNAs in plasma samples from women with advanced stage vs. early/intermediate, benign breast conditions (BBC) group and controls. Significant value in Kruskal–Wallis and Dunn’s post test (±SEM - standard error of the mean; ** *p* < 0.01; *** *p* < 0.01).

Finally, the miRNA expression levels were analyzed together to verify the strength of their combined use in plasma samples. Our results showed the combined amount of both miRNAs was able to differentiate patients with BC from control group (*** *p* < 0.001). However, there was no significant difference between the BC and BBC group (Figure 6a). In addition, the two miRNAs together were able to differentiate patients in advanced stage of BC from patients with early/intermediate stage of BC (** *p* < 0.01), advanced stage from BBC (* *p* < 0.05) and, lastly, advanced stage from control (*** *p* < 0.001). The other peer-compared groups showed no significant differences (Figure 6b).

### 3.3. miR-210 and miR-152 Are Stable in Bloodstream

**Figure 7:**
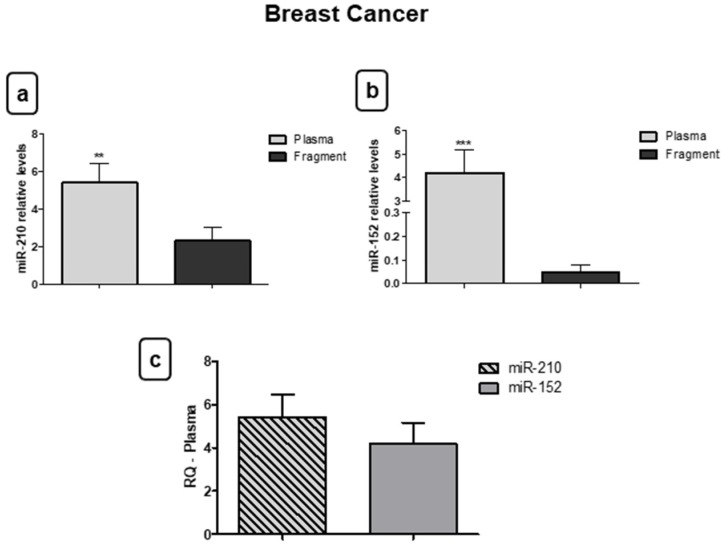
Both miR-210 and miR-152 are stable in blood. (**a**) For the breast cancer (BC) group, relative quantification showed that there was increased expression of miR-210 in plasma samples when compared with fragments. (**b**) The same occurred for miR-152; the relative quantification of this miRNA was higher in plasma samples compared to fragments. (**c**) There was no significant difference in circulating levels between miRNAs. Significant value in Kruskal–Wallis and Dunn’s post test (±SEM - standard error of the mean; ** *p* < 0.01; *** *p* < 0.01).

Subsequently, the differences in the miRNA expression in fragments and plasma samples were analyzed for the BC group. The results reveal that miR-210 (** *p* < 0.01) (Figure 7a) and miR-152 (*** *p* < 0.001) (Figure 7b) presented increased expression in plasma samples compared to the fragments, indicating that oncomiR, and even the tumor suppressor, have stabilizing mechanisms in the bloodstream. Although both miRNAs were increased in plasma samples compared to the fragment, there was no significant difference in circulating levels between them (Figure 7c).

### 3.4. Expression of miRNA Target Proteins by Immunohistochemistry

**Figure 8:**
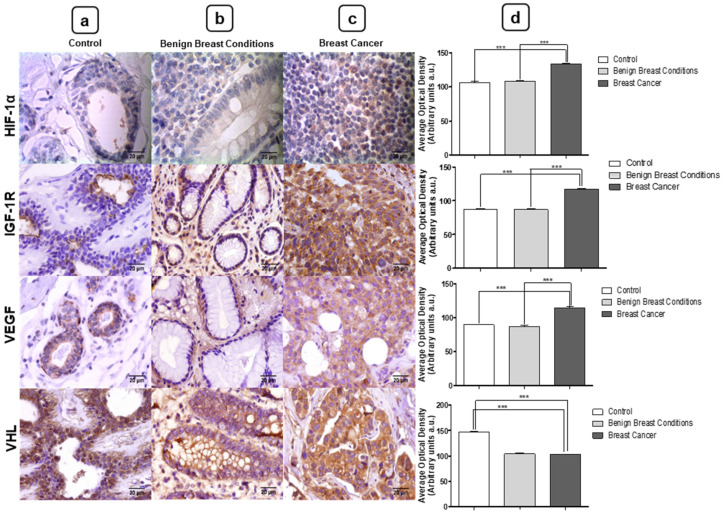
(**a**) Photomicrographs of (**a**) control, (**b**) benign breast conditions (BBC), and (**c**) breast cancer (BC). (**d**) Evaluation of protein expression in the three different groups. Hypoxia-inducible factor 1-alpha (HIF-1α), insulin-like growth factor 1 receptor (IGF-1R), and vascular endothelial growth factor (VEGF) showed a significant increase in the BC group compared to the group with BBC and the control group. Contrastingly, Von Hippel-Lindau (VHL) showed a significant decrease in the BC group compared to the group with BBC and the control group. Significant value in Kruskal–Wallis and Dunn’s post test (±S.E.M - Standard Error of the Mean; *** *p* < 0.001 vs. the control group; *** *p* < 0.001 vs. the BBC group). Magnification of 40×. Bar: 20 μm.

To confirm the action of miR-210 and miR-152 on the angiogenesis pathway, we verified the expression of the target proteins of both miRNAs by immunohistochemistry. All proteins showed cytoplasmic marking. Evaluation of protein expression by immunohistochemistry revealed increased levels of HIF-1α (*** *p* < 0.0001), IGF-1R (*** *p* < 0.0001), and VEGF (*** *p* < 0.0001) proteins in BC tissues compared to the control group. In addition, the proteins also showed increased expression in BC samples compared to the BBC group (HIF-1α *** *p* < 0.0001, IGF-1R *** *p* < 0.0001, and VEGF *** *p* < 0.0001). With respect to VHL, this protein showed decreased expression in BC samples when compared to the control group (*** *p* < 0.0001) and BBC group (*** *p* < 0.0001) (Figure 8).

### 3.5. Evaluation of miR-210 and miR-152 as Potential Diagnostic and Staging Biomarkers

**Figure 9:**
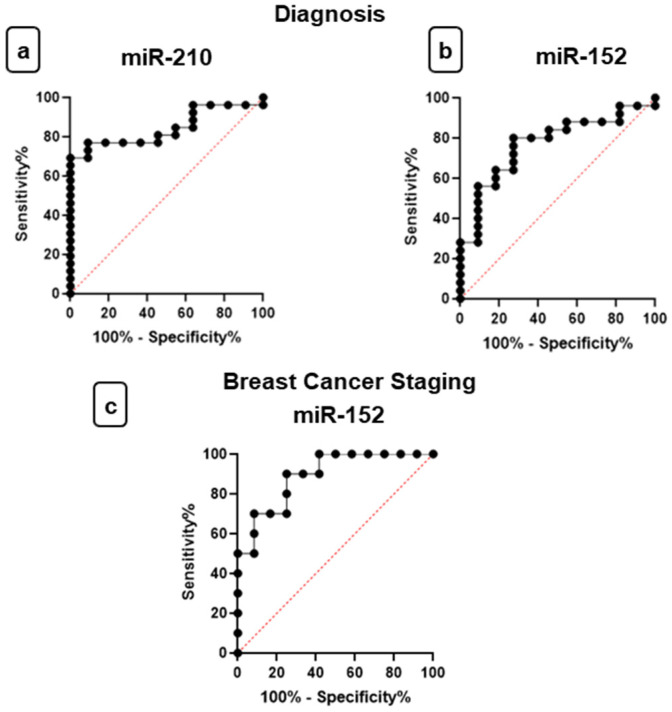
Receiver operating characteristic (ROC) curves investigate the diagnostic and staging power of miRNAs. (**a**) Plasma levels of miR-210 and (**b**) miR-152 discriminate breast cancer (BC) patients from healthy individuals—area under the curve (AUC) = 0.8427 and AUC = 0.709, respectively; cut-off value of 1.690 and of 0.252, respectively. Plasma levels of miR-152 (**c**) discriminate advanced stage of BC patients from early/intermediate stage of BC patients (AUC = 0.8917; cut-off value of 2.573).

Analysis of the ROC curve was used to determine miR-210 and miR-152 in plasma as a diagnostic biomarker to differentiate patients with BC from healthy individuals. The AUC for miR-210 for women with BC compared to the control group was 0.8427 (0.7174 to 0.9680) (*p* = 0.0011) with optimal sensitivity and specificity values of 76.9% and 90.9%, respectively, to the cut-off value of 1.690 as well as a positive predictive value of 95.24% and a negative predictive value of 62.44% (Figure 9a).

The AUC for miR-152 in plasma expression for women with BC compared to the control group was 0.7709 (0.6095 to 0.9324) (*p* = 0.0105) with optimal sensitivity and specificity values of 80% and 72.7%, respectively, to the cut-off value of 0.252 as well as a positive predictive value of 86.92% and a negative predictive value of 61.58%. (Figure 9b). Therefore, miR-210 and miR-152 expression in plasma samples have appropriate sensitivity and specificity to differentiate women without BC from women with BC (Figure 9).

ROC curve analysis was also performed to investigate the role of mir-152 in plasma as a staging biomarker for distinguishing advanced stage of BC patients from early/intermediate stage BC patient. On RT-qPCR, miR-210 did not show significantly different levels between women with early/intermediate stage of BC and women with advanced stage. The miR-152 showed good results in ROC analysis for women with advanced BC compared to women with early/intermediate stages of BC. The AUC was 0.8917 (0.7598 to 1.0000) (*p* = 0.0019) with optimal sensitivity and specificity values of 90% and 75%, respectively, to the cut-off value of 2.573 as well as a positive predictive value of 73.03% and a negative predictive value of 89.98% (Figure 9c).

**Figure 10:**
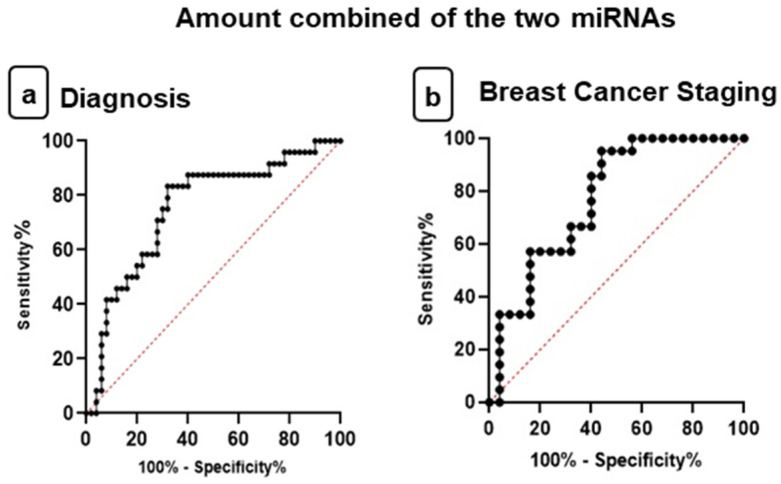
Receiver operating characteristic (ROC) curves investigate the diagnostic and staging power of amount combined of the miRNAs. (**a**) Plasma levels of miR-210 with miR-152 discriminate breast cancer (BC) patients from healthy individuals—area under the curve (AUC) = 0.7542; cut-off value of 0.9571. (**b**) Plasma levels of miR-210 with miR-152 discriminate advanced stage of BC patients from early/intermediate stage of BC patients (AUC = 0.7733; cut-off value of 2.660).

Ultimately, the ROC curve was used with the combined amount of these two miRNAs to determine whether together they could be diagnostic and prognostic biomarkers for BC (Figure 10).

The AUC for both miRNAs in plasma expression for women with BC compared to the control group was 0.7542 (0.6345 to 0.8739) (*p* = 0.0004) with optimal sensitivity and specificity values of 83.33% and 68.0%, respectively, to the cut-off value of 0.9571 as well as a positive predictive value of 89.47% and a negative predictive value of 55.51% (Figure 10a). Thus, the combined amount expression in plasma samples has appropriate sensitivity and specificity to differentiate the control group to women with BC.

ROC curve analysis was also performed to investigate the role of miR-210 with miR-152 in plasma together as staging biomarkers for distinguishing advanced stage from early/intermediate stage of BC. The AUC was 0.7733 (0.6382 to 0.9084) (*p* = 0.0016) with optimal sensitivity and specificity values of 85.71% and 60%, respectively, to the cut-off value of 2.660 as well as a positive predictive value of 83.29% and a negative predictive value of 64.33% (Figure 10b). Therefore, miRNAs together showed good results in the analysis of the ROC curve as prognostic biomarkers. However, the AUC showed that miRNAs individually are better diagnostic and prognostic biomarkers for BC.

## 4. Discussion

This study aimed to identify and validate two promising circulating miRNAs as biomarkers for diagnosis and staging of BC. In the clinical routine, a useful biomarker is still not available that is able to detect early malignant breast tumors and also predict the clinical evolution of the disease, with the advantage of being minimally invasive and routinely quantified [21]. In addition to the benefits of liquid biopsy, studies show that miRNAs are stable in biological fluids, and therefore have been investigated as potential circulating biomarkers [9]. Thus, the well-consolidated miRNAs in BC were selected to verify if they have good potential as circulating biomarkers.

miR-210 has been reported as one of the most consistent miRNAs for this neoplasm [22]. In this study, the results showed increased expression levels of miR-210 in tumor fragment samples. Thakur et al. also observed increased expression of this miRNA in tissue and cell lines of BC (MDA-MB-231 and MCF-10) [23]. Subsequently, the oncomiR-210 target protein expressions involved in the angiogenesis pathway were validated. In BC samples, this study found increased levels of HIF-1α expression and decreased levels of VHL. In neuroblastoma tumors, Ognibene et al. also observed that most hypoxic tumors present high expression of HIF-1α, which is a prognostic indicator to stratify high-risk patients [24]. In renal carcinoma cells, densitometry analysis revealed that the protein expression of VHL in cancerous tissue was lower when compared to normal adjacent tissue of the patients [25].

Another miRNA addressed in this study was miR-152. This is considered a tumor miRNA suppressor well validated in several types of neoplasms, including BC [16,26]. In this study, decreased levels of miR-152 expression were observed in tumor fragments. In addition to these results, Wen et al. found that miR-152 expression levels were significantly lower in BC fragments than in non-cancerous tissues [27]. Furthermore, Ge et al. investigated the role of this miRNA in the initiation and progression of BC and found that miR-152 expression was significantly reduced in BC fragments and in cell lines [19]. MiR-152 acts on the angiogenesis pathway as a tumor suppressor, leading to IGF-1R blockade [16]. In agreement with our findings, studies have observed the overexpression of IGF-1R in BC samples, and this may influence the heterogeneity of molecular subtypes [28].

Both cascades of miR-210 and miR-152 have a final interference on the VEGF protein, which culminates in the development of new blood vessels for the tumor microenvironment [29]. In this study, the expression of VEGF in BC samples was validated. As with our results, previous studies have shown an increase of this protein in BC and also in other neoplasms [30,31].

After verifying the expression of these miRNAs and their target proteins in BC fragments, we questioned whether they could be potential circulating biomarkers for this neoplasm. According to Chen et al. [32], the source of circulating miRNAs in cancer patients is controlled by the following three mechanisms: (i) passive miRNA leakage from tumor cells, (ii) active secretion via microvesicles, and (iii) active secretion using an RNA-binding protein-dependent pathway, without microvesicles. Nonetheless, according to these authors, the miRNAs from sources (i) and (ii) have stability in the blood circulation, despite the presence of ribonucleases.

In the specific case of miR-210, this study found increased levels in the plasma of patients with BC, which coincides with the findings in the fragments. In agreement with these results, Madhavan et al. observed that miR 210 presented high levels in plasma samples from women with BC, and its expression was associated with the appearance of metastasis for up to two years after confirmation of the diagnosis [33]. Markou et al. also found significantly elevated plasma miR-210 levels in women with metastatic BC compared to healthy women [34]. Moreover, Qattan et al., comparing the expression levels of plasma miRNAs from women with BC and available RNASeq data from breast tumor tissue, also found that miR-210 showed increased levels of expression in both samples [35].

Cancer cells secrete various types of humoral factors in their tumor microenvironment and, among them, are extracellular vesicles (microvesicles and exosomes). Currently, evidence suggests the importance of communication between cancer cells and their microenvironment by releasing membrane exosomes that can fuse with nearby cells [36]. According to Adouh et al., circulating blood exosomes are the main contributing factors involved in the horizontal transfer of malignant characteristics to the target cells [37]. These horizontal molecular transfers can modulate cell-signaling pathways, such as the miR-210 pathway of angiogenesis. It is concluded, therefore, that the increased levels of miR-210 in plasma may reflect molecular changes in the cells from which they are derived [38].

Our results showed increased expression of circulating miR-152 in the plasma of women with BC. This was an interesting finding in this study because, as already described, miR-152 has a suppressive action on fragments of breast neoplasms. In the literature, there is only one study involving miR-152 and blood samples from patients with BC. The authors also observed increased plasma levels of miR-152 in patients with the disease and in patients with benign lesions compared to the control group. Furthermore, the increased expression of plasma miR-152 levels was also observed in patients with lung and colon cancer when compared to the control group [39]. Another study demonstrated that an increased level of circulating miR-152 was found in patients with bladder cancer, which was related to tumor recurrence of invasive bladder cancer [40]. In addition, Lin et al. showed that the tumor suppressor miR-34a was upregulated in esophageal cancer and concluded that this miRNA can serve as a potential biomarker in the detection of the early stages of this neoplasia [41]. Moreover, Qattan et al. observed an inverse pattern between plasma and tissue levels of patients with BC triple negative for let-7b, miR-29c, and miR-22. In all cases, plasma miRNA levels were higher than tissue levels [35].

According to Kosaka et al., negatively regulated miRNAs in cancer tissues are supplied during the initial stage of tumorigenesis by the surrounding cells that provide exosomes containing the decreased miRNAs. However, if the surrounding cells fail to meet demand, the cancer cells end up entering an advanced oncogenic stage. Thus, microRNAs secreted by exosomes can be useful for the maintenance and surveillance system against cancer progression [42].

In some cases, the exosomal mechanism can be used by metastatic cells to eliminate miRNAs with suppressive functions. For example, one study showed that miRNAs in the let-7 family, which preferentially act as a tumor suppressor, showed increased levels of expression in metastatic gastric cancer cells compared to non-metastatic parental cells. Therefore, the authors concluded that the let-7 miRNA family is rich in exosomes from a metastatic gastric cancer [43]. Yet, another study showed that tumor suppressor miR-23b, highly secreted by exosomes from metastatic cells, showed decreased levels in lymph node metastasis compared to primary tumors. Thus, the exosome-mediated secretion of tumor suppressor miRNA is selected during tumor progression as a mechanism to coordinate the activation of a metastatic cascade [44].

Therefore, exosomes play an important role in communicating with the tumor microenvironment, since cancer cells can modulate the stromal environment in their favor, or even surrounding cells can try to stop the evolution of malignancy by “delivering” protective miRNAs [45]. Many studies show that circulating miRNAs packaged by extracellular vesicles are protected from the degradation action of ribonucleases and, therefore, present high levels in the bloodstream. In this way, so as not to be degraded in the bloodstream, secretory vesicles carry circulating miRNAs [46,47].

In summary, we hypothesized that the discrepancies in the expected miR-152 expression in plasma and tissue fragments occur because circulating miRNAs packaged by extracellular vesicles are protected from the degradation action of ribonucleases and, thus, present high levels in the bloodstream. Despite the advances made regarding miR-152, further studies are needed to determine which regulatory mechanisms for miRNAs are secreted by exosomes, especially with regard to tumor suppressor miRNAs present in biological fluids. This will provide enormous opportunities for cancer-targeted therapies in the near future.

In order to establish the value of miRNAs as circulating biomarkers of diagnosis and staging, ROC curves were constructed and the AUC calculated. Hosmer and Lemeshow defined a rule that studies follow to identify good results in the ROC curves, based on respective AUC values: “0.5 = this suggests no discrimination, so we might as well flip a coin; 0.5–0.7 = we consider this poor discrimination, not much better than a coin toss; 0.7–0.8 = acceptable discrimination; 0.8–0.9 = excellent discrimination and >0.9 = outstanding discrimination” [48].

In the present study, results showed that miR-210 in plasma samples have excellent discrimination to be used as a biomarker of diagnosis, according to the AUC of the ROC curve comparing the control group and BC. With the same objective, other studies obtained similar results. Markou et al. evaluated miR-210 by ROC curve analysis and concluded that it was a valuable biomarker for discriminating patients from healthy individuals, with AUCs of 0.959 (95% Confidence Interval (CI) = 0.917–1.000, *p* < 0.0001) [34].

Despite the unexpected increased expression of miR-152 in women with BC, ROC curve analysis demonstrated that miR-152 expression in plasma samples has an acceptable discrimination to be used as a biomarker of diagnosis. Furthermore, miR-152 has shown greater sensitivity compared to miR-210, which demonstrated its greater capacity in the screening of BC. In contrast, miR-210 was shown to be the most specific in the analysis, which could indicate a greater capacity, compared to the miR-152, to confirm the diagnosis of BC, with lower false-positive results. In the same way, other authors obtained similar results. Chen et al. observed significant upregulated miR-152 expression with a 2.91-fold change in BC patients (*p* = 0.00275), when compared to normal controls. Furthermore, they evaluated miR-152 by ROC curve analysis and concluded that it was a valuable plasma biomarker for discriminating patients with BC and BBC from normal controls [39].

To investigate the role of miR-152 in plasma staging biomarkers, the values of AUC showed that miR-152 plasma expression has an outstanding discrimination to differentiate early/intermediate and advanced stage of BC patients. Furthermore, Dasari et al. evaluated that the level of miR-152 in peripheral blood was higher in patients with high-grade cervical intraepithelial neoplasia (CIN) compared with those with low-grade CIN, which demonstrates the prognostic importance of this miRNA in the carcinogenic process [49].

## 5. Conclusions

In conclusion, analysis of miRNA expression in the fragments showed increased miR-210 and decreased miR-152 in BC, also validated by the expression of their target proteins involved in angiogenesis. Among miRNAs, miR-210 had a potential as a diagnostic biomarker and miR-152 presented a potential as diagnostic and staging biomarkers. The combination of the two miRNAs has the potential to be used as diagnostic and prognostic biomarkers in BC, but not better than the miRNAs used individually. These results may contribute to the use of these miRNAs as promising circulating markers in addition to the benefit of being detected by liquid biopsy. In the future, it is also expected that the miRNAs may be potential therapeutic targets, contributing to advances in personalized medicine.

## Data Availability

The datasets used and/or analysed during the current study are available from the corresponding author on reasonable request.

## Supplementary Materials

The following are available online at https://www.mdpi.com/2075-4426/11/1/31/s1, Table S1: Description of microRNAs, Table S2: Primary antibodies used in immunohistochemical techniques and their respective specifications, Figure S1: Photomicrographs of the positive and negative controls of the target proteins during the immunohistochemistry reaction. Magnification of 40X. Bar: 20 μm.
