# Supplementary material for: miR-210 and miR-152 as Biomarkers by Liquid Biopsy in Invasive Ductal Carcinoma
Source: J Pers Med. 2021 Jan 6;11(1):31. doi: 10.3390/jpm11010031 (PMC7825421; doi:10.3390/jpm11010031)

MiR-210 and miR-152 as Biomarkers by Liquid Biopsy in Invasive Ductal Carcinoma

Beatriz Camargo Lopes[1,2], Fabrício Valiante Ventura[2], Jéssica Gisleine de Oliveira[2,3], Cristine Zampieri Braga[2], Edson Mitsuro Kato-Junior[2], Newton Antonio Bordin-Junior[3], Debora Aparecida Pires de Campos Zuccari[1,2,3*]

SUPPLEMENTARY MATERIALS

Table S1. Description of microRNAs.

miR-210-3p	
Chromosome Location	Chr.11 - 568112-568171 [-]<br>on *UCSC Ensembl*
Mature miRNA Sequence	CUGUGCGUGUGACAGCGGCUGA
Stem Loop	ACCCGGCAGUGCCUCCAGGCGCAGGGCAGCCCC UGCCCACCGCACACUGCGCUGCCCCAGACCCACU GUGCGUGUGACAGCGGCUGAUCUGUGCCUGGGC AGCGCGACCC

miR-152-3p	
Chromosome Location	Chr. 17 - 48037161 - 48037247 [-]<br>*on Build* GRCh38
Mature miRNA Sequence	UCAGUGCAUGACAGAACUUGG
Stem Loop	UGUCCCCCCCGGCCCAGGUUCUGUGAUACACUC CGACUCGGGCUCUGGAGCAGUCAGUGCAUGACA GAACUUGGGCCCGGAAGGACC

Source: miRBase (http://www.mirbase.org)

Table S2. Primary antibodies used in immunohistochemical techniques and their respective specifications.

Antibody	Company	Clone	Diluition IHC	Marking	Positive Control
**HIF-1α**	*Santa Cruz Biotechnology, Dallas, TX, USA*	H1alpha67	1:25	Cytoplasmatic	Human breast Carcinoma

**VHL**	*ABCAM, Cambridge, UK*	1E1	1:200	Cytoplasmatic	Kidney
**IGF-1R**	*Sigma-Aldrich, St. Louis, MO, USA*	C-terminal	1:50	Cytoplasmatic	Liver
**VEGF**	*Santa Cruz Biotechnology, Dallas, TX, USA*	A-20	1:300	Cytoplasmatic	Liver

Figure S1. Photomicrographs of the positive and negative controls of the target proteins during the immunohistochemistry reaction. Magnification of 40×. Bar: 20 μm.

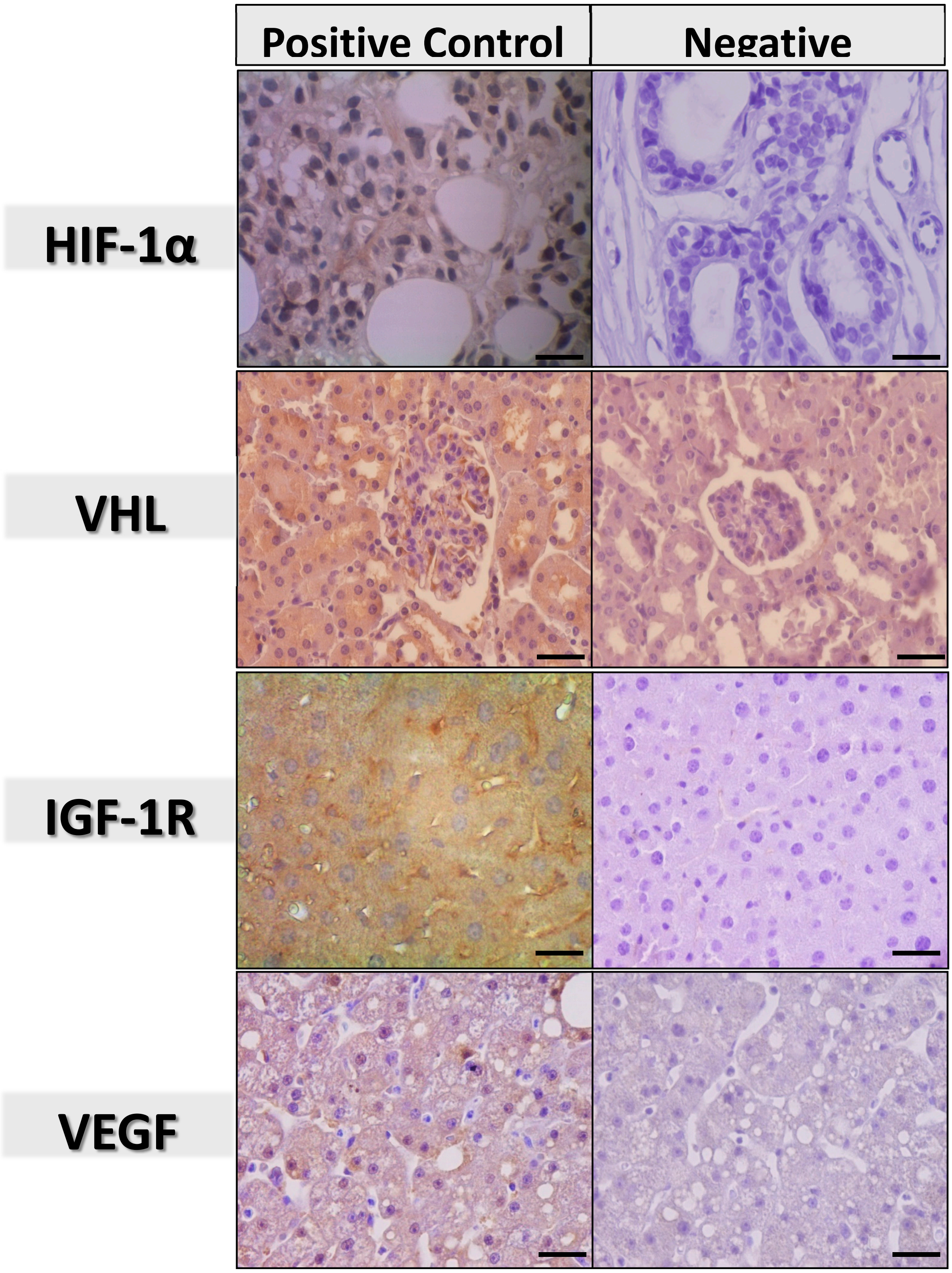

Supplement: Supplementary file 1 [file jpm-11-00031-s001.pdf]
